# Population Genomics of the “Arcanum” Species Group in Wild Tomatoes: Evidence for Separate Origins of Two Self-Compatible Lineages

**DOI:** 10.3389/fpls.2021.624442

**Published:** 2021-03-19

**Authors:** Ana M. Florez-Rueda, Mathias Scharmann, Morgane Roth, Thomas Städler

**Affiliations:** Plant Ecological Genetics, Institute of Integrative Biology and Zurich–Basel Plant Science Center, ETH Zurich, Zurich, Switzerland

**Keywords:** mating systems, population genomics, self-fertilization, nucleotide diversity, *Solanum*, species delimitation, *S*-locus

## Abstract

Given their diverse mating systems and recent divergence, wild tomatoes (*Solanum* section *Lycopersicon*) have become an attractive model system to study ecological divergence, the build-up of reproductive barriers, and the causes and consequences of the breakdown of self-incompatibility. Here we report on a lesser-studied group of species known as the “Arcanum” group, comprising the nominal species *Solanum arcanum*, *Solanum chmielewskii*, and *Solanum neorickii*. The latter two taxa are self-compatible but are thought to self-fertilize at different rates, given their distinct manifestations of the morphological “selfing syndrome.” Based on experimental crossings and transcriptome sequencing of a total of 39 different genotypes from as many accessions representing each species’ geographic range, we provide compelling evidence for deep genealogical divisions within *S. arcanum*; only the self-incompatible lineage known as “var. marañón” has close genealogical ties to the two self-compatible species. Moreover, there is evidence under multiple inference schemes for different geographic subsets of *S. arcanum* var. marañón being closest to *S. chmielewskii* and *S. neorickii*, respectively. To broadly characterize the population-genomic consequences of these recent mating-system transitions and their associated speciation events, we fit demographic models indicating strong reductions in effective population size, congruent with reduced nucleotide and *S*-locus diversity in the two independently derived self-compatible species.

## Introduction

The causes and consequences of evolutionary transitions in mating systems have played prominent roles in both fundamental and applied research on flowering plants. Of particular interest is the frequent transition from ancestral self-incompatibility (SI) to derived self-compatibility (SC), which has been investigated from molecular, evolutionary, and ecological vantage points (Stebbins, 1957; Gottlieb, 1977; Lloyd, 1992; de Nettancourt, 2001; Castric and Vekemans, 2004; Busch and Schoen, 2008; Igic et al., 2008; Sicard and Lenhard, 2011; Wright et al., 2013; Pannell et al., 2015). A related issue of high interest to evolutionary biologists is whether a shift in mating system (SI-to-SC) promotes, or even initiates, the evolutionary divergence of two or more independent lineages, eventually resulting in (at least partial) reproductive isolation and speciation (Cutter, 2019).

The latter aspect has been thoroughly investigated in the genus *Capsella*, where the recently diverged self-compatible *Capsella rubella* was shown to be derived from the obligately outcrossing *Capsella grandiflora* (Foxe et al., 2009; Guo et al., 2009; Brandvain et al., 2013; Bachmann et al., 2019). With such transitions to self-fertilization concomitant with rapid speciation, demographic bottlenecks resulting in low levels of nucleotide diversity are plausible and have been documented in *Capsella* (Foxe et al., 2009; Guo et al., 2009) as well as in *Mimulus* (Brandvain et al., 2014). The population genomic consequences of such mating-system transitions, however, are still under-explored and have only been extensively studied in few cases (Barrett et al., 2014; Laenen et al., 2018; Cutter, 2019).

There has been a long-standing debate about the selective advantages and disadvantages of self-fertilization, with “automatic selection” (Fisher, 1941) and “reproductive assurance” (Baker, 1955) as the prime candidates for the genetic and ecological agents underpinning the selection of self-fertilization (Busch and Delph, 2012). Assumed long-term disadvantages of a largely selfing mating system, such as limited adaptive potential (e.g., Noël et al., 2017), are at the core of viewing prolonged selfing as an “evolutionary dead end” (Stebbins, 1957; Takebayashi and Morrell, 2001; Igic and Busch, 2013). Molecular data on the extent of within-population diversity between outcrossing and selfing lineages have been proposed as one way to discriminate between the two main hypotheses for the short-term advantage of selfing (Schoen et al., 1996; Busch and Delph, 2012). However, the realization that selection on linked neutral polymorphisms in regions of low recombination (with genome-wide effects in highly selfing populations; Cutter and Payseur, 2013) will also diminish variation—just like demographic bottlenecks proposed under situations of reproductive assurance—implies that empirical tests might often be inconclusive (Barrett et al., 2014). Moreover, metapopulation dynamics as an additional factor above the scale of local/regional populations might either increase or decrease levels of variation at the species-wide level (Ingvarsson, 2002).

In the plant family Solanaceae (the “nightshade” family), SI is of the gametophytic type and controlled by a single *S*-locus coding for extracellular S-RNases expressed in pistils and *S*-locus F-box proteins (SLFs) expressed in pollen (Kubo et al., 2015; Fujii et al., 2016; Markova et al., 2016, 2017, and references therein). Comparative evolutionary research in Solanaceae has suggested that the loss of SI is likely irreversible (Igic et al., 2006; Goldberg et al., 2010), which is consistent with the molecular changes underlying the breakdown of SI (i.e., mutations disabling the pistil-expressed S-RNase protein and possibly gain-of-function mutations in SLFs; Kubo et al., 2015; Markova et al., 2017).

Wild tomatoes (*Solanum* section *Lycopersicon*) are well-suited to address these issues because they comprise a suite of closely related species with diverse mating systems (Peralta et al., 2008; Nakazato et al., 2010), patterns of species divergence (Städler et al., 2005, 2012) and life-history characteristics (Tellier et al., 2011a, b). In particular, ancestral SI has been lost repeatedly, with entire nominal species being self-compatible like in the red-fruited tomato clade (e.g., Spooner et al., 2005), or peripheral populations of otherwise self-incompatible taxa having acquired SC (Markova et al., 2016; Broz et al., 2017). On the other hand, basic questions about the number of evolutionarily independent lineages linger, and the group’s taxonomy continues to be debated (Peralta et al., 2005, 2008; Aflitos et al., 2014; Labate et al., 2014; Pease et al., 2016).

Here we focus on the so-called “Arcanum” species group, comprising the two self-compatible green-fruited species *Solanum chmielewskii* and *Solanum neorickii* as well as the self-incompatible *Solanum arcanum* (Supplementary Figure 1). Jointly with other lineages that formerly were part of the highly diverse *Solanum peruvianum* sensu lato, the latter was recognized as a separate species by Peralta et al. (2005, 2008). Recent genome-wide molecular surveys failed to unambiguously place *S. arcanum* phylogenetically, in large part due to sequencing only one or two individuals (Aflitos et al., 2014; Pease et al., 2016). Genome-wide sequence data are compatible with paraphyly of *S. arcanum* as currently circumscribed, and seminal morphological and crossing studies found strong postzygotic barriers between geographically separated groups of populations (Rick, 1963, 1986) that are currently all treated as *S. arcanum*. A single accession of *S. arcanum* is known to be self-compatible (Kowyama et al., 1994; Royo et al., 1994b), with two others suspected of segregating for SI/SC (Tomato Genetics Resource Center^1^ (TGRC)).

Recent analyses of molecular defects in *S*-locus genes and other components of the SI/SC reaction in the Arcanum species group have accrued strong evidence for independent origins of SC in *S. chmielewskii* and *S. neorickii* (Markova et al., 2017), and thus an evolutionary scenario different from that espoused by Rick et al. (1976) when these two taxa were originally described and compared. Based on the strong morphological selfing syndrome and apparent lack of allozyme polymorphism in *S. neorickii* (“*NEOR*”), Rick et al. (1976) argued for its divergence from *S. chmielewskii* (“*CHMI*”) after a single, earlier transition to SC in their common ancestor. Rick et al. (1976) also demonstrated low seed set in reciprocal crosses and extremely low germination of F2 hybrid seeds, consistent with the absence of hybrids in regions of sympatry; Rick et al. (1976) did not speculate about the affinity of both self-compatible taxa to other wild tomato species known at the time. As is true for all wild tomato species including *S. arcanum* (“*ARCA*”), the two self-compatible taxa are short-lived perennials with no obvious divergence in life history. They are slightly diverged in their abiotic preferences and altitudinal range (Nakazato et al., 2010) but were also found to occur in sympatry in *CHMI*’s more restricted range (Rick et al., 1976). To our knowledge, there are no detailed field-based studies of their ecology or demography.

Here, we investigate the phylogenomics and patterns of genomic variation in relation to outcrossing–selfing transitions in the Arcanum species complex of wild tomatoes, based on range-wide sampling of available accessions and transcriptome sequencing, yielding approximately 5 million genome-wide SNPs after the initial filtering steps. In particular, our study has the following objectives:

(i) Can the classic crossing data (Rick, 1963, 1986) documenting postzygotic barriers within what is now considered *ARCA* be substantiated and cogently interpreted using our molecular data? (ii) to clarify the phylogenomic relationships of the two self-compatible species vis-à-vis the outcrossing *ARCA* [*CHMI* and *NEOR* may have a single common origin as advocated by Rick et al. (1976) or two independent origins as suggested by the data of Markova et al. (2017)]; (iii) to infer the demographic histories of *CHMI* and *NEOR* in the context of the most likely sequence of population splits (tree topology) yielding the extant lineages; (iv) to leverage patterns and levels of nucleotide diversity in *CHMI* and *NEOR* compared to those in their inferred most closely related outcrossing sister lineage(s) to ascertain modes of selection for self-fertilization and the forces reducing genome-wide variation; and (v) to complement the genome-wide data with those from *S*-locus components that ought to show marked loss of allelic diversity in the wake of SI-to-SC transitions.

## Materials and Methods

### Sampling Scheme and Reciprocal Crosses

**FIGURE 1:**
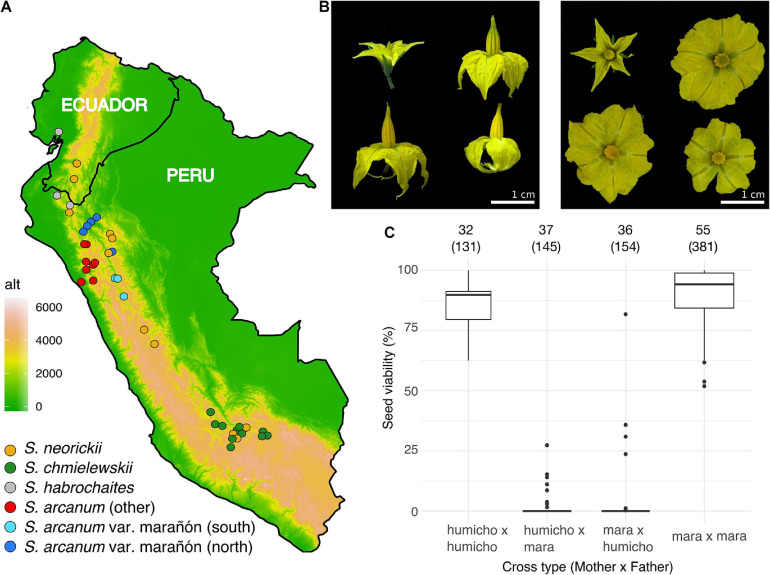
Plant sampling, floral diversity, and complex patterns of hybrid seed failure. **(A)** Map of Peru and Ecuador indicating the geographic origin of all 42 sequenced accessions and their taxonomic assignment (assignment within *S. arcanum* reflects our molecular data). Colored dots approximate the location of each TGRC accession. **(B)** Examples of corolla size/shape, anther cone, and stigma exsertion. Clockwise from top left, each panel features *NEOR* (LA1319), *MARA* (LA2185, *MARA-N*), *MARA* (LA1032, *MARA-S*), and *CHMI* (LA1316). Note that there is marked morphological variation within and between accessions for the latter three groups. **(C)** Proportion of viable seeds obtained from reciprocal crosses within and among subgroups of *ARCA*; “humicho” comprises the non-*MARA S. arcanum* with the exception of LA0378, LA0441, and LA1984 (full data are in Supplementary Figure 2B). Numbers atop the graph refer to the number of independent crosses, with the number of fruits sampled in parentheses.

Plants were grown from individual seeds obtained from the TGRC (University of California, Davis, CA, United States; see text footnote 1). Within the limits of accessions that can be obtained from the TGRC, we attempted to have each lineage represented by accessions spanning as much of their known geographic range as possible, so as to implement geographically scattered sampling (Städler et al., 2009). In addition, in view of the unsettled taxonomy and delimitation of wild tomato lineages that were formerly considered to be part of *S. peruvianum* sensu lato (Rick, 1986; Peralta et al., 2005, 2008), we included equal numbers of *S. arcanum* var. marañón (“*MARA*”) and those representing other subgroups within the nominal *S. arcanum* (Figure 1A and Supplementary Figure 2A). Three individuals of *Solanum habrochaites* were included to serve as the outgroup in phylogenomic analyses. Supplementary Table 1 lists all 42 sequenced accessions and also includes data on mapping success of RNA-seq reads to the tomato reference genome (The Tomato Genome Consortium, 2012).

Plants were grown in individual pots in an insect-free greenhouse at the Lindau-Eschikon plant research station of ETH Zurich. Details of plant care, light, temperature and humidity conditions can be found in Roth et al. (2018). Scoring of seed viability following crosses within *ARCA* was motivated by Rick’s seminal studies of crossing relationships, performed at various times when the number of relevant accessions housed by the TGRC was more limited (Rick, 1963, 1986). Technical aspects of our crossing procedures were detailed in Roth et al. (2018). Briefly, we hand-pollinated freshly opened flowers of focal plants with pollen from another individual (reciprocally where possible) on various dates, resulting in several fruits for each pair of individuals for later harvest. Ripe fruits were harvested after 60 days and all seeds were counted and visually categorized as either “viable” or “inviable” (Supplementary Table 2). Viable seeds had a plump appearance and contained a well-developed embryo, while inviable seeds were flat in appearance and of variable size, depending on cross directionality. These systematic defects of normal seed development, due to endosperm failure, characterize many hybrid crosses among wild tomatoes and many other closely related species of flowering plants (e.g., Baek et al., 2016; Florez-Rueda et al., 2016; Oneal et al., 2016; Roth et al., 2019; Coughlan et al., 2020; Städler et al., 2021). Germination tests with F1 seeds obtained in other cross combinations have shown that visual seed inspection is a very good proxy for actual germination ability (Roth et al., 2018), as has also been observed in *Mimulus* (Sandstedt et al., 2020).

### Sequencing and Genotyping

RNA was extracted from unopened flower buds with RNeasy Plant Mini Kit from QIAGEN and RNAseq libraries were prepared using Illumina’s TruSeq^®^ RNA Sample Preparation Kit. Libraries were sequenced on an Illumina HiSeq2000 at the ETH Department of Biosystems Science and Engineering, Basel. Raw reads were mapped to the cultivated tomato reference genome (The Tomato Genome Consortium, 2012) using the Build SL3.0 genome assembly and the ITAG3.20 annotation via STAR implemented in SUSHI (Hatakeyama et al., 2016). We filtered the read alignments with samtools view and retained only those with high quality and only primary alignments (i.e., uniquely aligned reads). Variant sites were called using samtools and bcftools (Danecek et al., 2011). The resulting variant call file (vcf) was filtered for high-quality biallelic sites present in at least 80% of the samples via vcftools (Danecek et al., 2011). We thus recovered 5,530,891 variant sites (SNPs) across our 42 samples. This data was used as a starting point for the subsequent analyses and was subject to further filtering steps, as described below.

### Principal Component Analyses

We first explored patterns of genome-wide molecular relationships between the samples by performing Principal Component Analysis (PCA) in PLINK (Purcell et al., 2007), filtering for an analysis of all samples excluding the outgroup and a second analysis of only the two self-compatible species *CHMI* and *NEOR*, as well as *MARA*.

### Phylogenomic Analyses

We used two complementary approaches for phylogenetic inference: (i) maximum likelihood (Stamatakis, 2014) applied to a concatenated supermatrix and (ii) quartet-based gene tree reconciliation (Mirarab and Warnow, 2015). We generated per-gene alignments for all samples by applying the variants to the reference genome with bcftools consensus, and using the reference genome annotation. In this step, we also masked any low-coverage regions (fewer than six reads per site per sample) in a sample-specific manner, identifying such regions from the .bam read alignment files with samtools (Li et al., 2009). In this manner, we handled variant and invariant sites consistently so as to avoid ascertainment bias. We removed sites within the alignments missing in >50% of the samples per column, and retained only genes with a minimum of 200 bp alignment length and all 42 samples being present. Finally, the 7,343 retained gene alignments were concatenated to a supermatrix of 10,829,556 columns. A maximum-likelihood phylogeny was estimated for the concatenated supermatrix using RAxML (Stamatakis, 2014) with the substitution model GTRCAT and SH-like support values.

For quartet-based estimation of a species tree, we first estimated gene trees for each of the 7,343 gene alignments using RAxML with the GTRCAT substitution model, and then applied ASTRAL v4.10.9 (Mirarab and Warnow, 2015) to these gene trees. Quartets are subtrees of four taxa that are extracted from the gene trees, and the ASTRAL quartet support values of a bi-partition represent the proportion of quartets agreeing with that particular bi-partition of the species tree. This approach thus enables quantification of levels of gene-tree incongruence with the underlying species tree, e.g., due to incomplete lineage sorting expected under the recent divergence times of our focal lineages. Furthermore, we applied the test for “hard” polytomies proposed by Sayyari and Mirarab (2018) to the species tree and the gene trees (ASTRAL v5.7.5). The same set of gene trees was visualized as a “cloudogram” with the function densiTree of the R package *phangorn* (R Development Core Team, 2014; Schliep, 2011).

### Demographic Inference

Using the program momi2 (Kamm et al., 2020), we characterized the demographic history of four extant lineages: *NEOR*, *CHMI*, and two geographic subgroups of *MARA* that we refer to as “*MARA-N*” and “*MARA-S*” (see section “Results”). Momi2 efficiently computes the expected multi-population site frequency spectrum (SFS) under demographic models, and can fit such models to the observed SFS using maximum likelihood. As input data for momi2, we used fourfold degenerate sites (minimum coverage of six reads) present in all 31 individuals from the four populations. The genomic coordinates of fourfold degenerate sites were detected by a custom script that classified all codons in the tomato reference genome (Build SL3.0) based on its coding gene annotation (ITAG3.20). The tomato genome harbors large, gene-poor pericentromeric regions (approximately 75% of the assembled reference genome) with extremely low levels of recombination and gene-dense chromosome arms with variable but generally high levels of recombination (The Tomato Genome Consortium, 2012). To avoid possible biases due to tight linkage, we restricted the sites to those from high-recombination, euchromatic genomic regions, as delimited by Demirci et al. (2017). Because an inbreeding mating system distorts the population SFS (e.g., Blischak et al., 2020), we sampled only one randomly chosen allele from the diploid genotypes of the self-compatible lineages *NEOR* and *CHMI* (i.e., pseudo-haploid sampling; see Koenig et al., 2019).

A vcf with SNPs and the coordinates of the filtered sites (in .bed format) was converted to a folded momi2 SFS with 100 blocks for bootstrapping. This momi2 SFS is 249,228 sites long, of which 7,591 are polymorphic (bi-allelic SNPs). The mutation rate was set to 7.5 × 10^–9^ per site per generation, corresponding to the best available estimate of spontaneous mutation rates in plants (Krasovec et al., 2018). In general, we considered only population sizes that were constant over time; hence, each extant and ancestral population was given a separate, unconstrained population size. For model choice, we compared momi2 models using the best of 25 maximum-likelihood optimizations per model, using the Akaike information criterion (AIC; Akaike, 1973). For the best model, we evaluated the robustness of parameter estimates by optimizations for 200 parametric bootstraps of the SFS.

### Population-Genomic Analyses

We used the vcf genotypes, the reference genome sequence and its annotation to calculate average nucleotide diversity π within groups, Tajima’s *D* statistic, and absolute nucleotide divergence (D_XY_) between groups (species and subgroups) in PopGenome (Pfeifer et al., 2014). To avoid the effects of missing data in population-genetic estimators, we used the suite of BEDTools options (Quinlan and Hall, 2010) to filter the gff annotation file and retained only CDS features covered for at least 75% of their length with a minimum coverage of six reads per sample in all 42 samples. The final gff input into PopGenome consisted of 13,841 CDS grouped into 3,722 genes. To explore possible variation of nucleotide diversity among two very different recombinational environments, we split our analyses into euchromatic and heterochromatic regions, following the delimitation described in Demirci et al. (2017). For chromosome-length visualization, we generated Manhattan plots of half of the 12 tomato chromosomes and plotted lines of smoothed conditional means of π in *MARA*, *NEOR*, and *CHMI*, using a generalized additive model.

### Seeded *de novo* Assembly of *S*-*RNase* and *SLF* Genes

To identify molecular elements of the SI system in our tomato samples, we followed a seeded *de novo* assembly strategy, because the tomato reference genome is lacking components of the SI system and represents only one of many possible haplotypes, rendering it too divergent for conventional sequence-read mapping. As seed sequences we used the 38 *S-RNase* and *SLF* alleles reported by Markova et al. (2016, 2017). We then extended the seed sequence collection by BLAST-searching the above sequences against GenBank nt restricted to *Solanum* section *Lycopersicon* (NCBI: txid49274) with an *e*-value threshold of 1e-5. Very long target sequences were excluded (i.e., chromosomes and genomic contigs). We downloaded the nucleotide coding sequences of the identified GenBank entries using Entrez Direct. This resulted in a final collection of 213 coding sequences of *S-RNase* and *SLF* alleles for seeded *de novo* assembly (Supplementary Table 3). While many of them were published (Chung et al., 1993, 1994, 1995; Rivers et al., 1993; Royo et al., 1994a, b; Kondo et al., 2002; Igic et al., 2007; Aoki et al., 2010; Miller and Kostyun, 2011; Li and Chetelat, 2015; Markova et al., 2016, 2017), several of these GenBank entries appear to be from unpublished studies.

Seeded, iterated *de novo* assembly was performed separately for each of our 39 transcriptome samples (disregarding those from *S. habrochaites*). In brief, BLAST databases from raw sequencing reads were searched for matches to the seed sequence collection under permissive criteria (*e*-value threshold 1e-5). Any matching raw reads were then subjected to *de novo* contig assembly with the RNA-seq assembly pipeline Trinity v2.9.1 (Grabherr et al., 2011) using standard parameters. In the second iteration, the contigs assembled by Trinity were added to the seed sequence collection, and again searched against the raw read databases for matches, followed by assembly in Trinity. The same process was repeated twice, amounting to a total of four iterations of raw read matching and *de novo* assembly. Only the longest “isoform” (sensu Trinity) of each assembled “gene” (sensu Trinity) was retained, minimizing the possibility that true alleles are confounded by splice variants and alternate assembly results. We used TransDecoder (Haas et al., 2013) to predict and retain the single, best scoring open reading frame per *de novo* sequence, and continued with only these predicted coding sequences. Subsequently, we reduced redundancy once again by clustering sequences within each sample with CD-HIT (Li and Godzik, 2006) at the threshold of 98% identity, retaining only the longest sequence per cluster.

Finally, all *de novo* sequences were BLAST-searched against the GenBank nt database (*e*-value 1e-5) to identify and exclude potential off-target sequences, defined as any sequences that did not have a Solanaceae *SLF* or *S-RNase* as their best match. For *SLF*s, we restricted the downstream analyses to *SLF-23*, one of several SLF paralogs in tomato genomes that has been demonstrated to be sufficient for the incompatibility reaction by transformations (Li and Chetelat, 2015) and likely played a role in the acquisition of SC in *NEOR* (Markova et al., 2017). The seed and *de novo* sequences for *SLF-23* and *S-RNase*s were aligned by the stop codon- and frameshift-aware tool MACSE v2.03 (Ranwez et al., 2018) with two refinement iterations. Nucleotide alignments were filtered for minimum column presence of 5% and phylogenies were built using RAxML v8.2.12 (Stamatakis, 2014) under the GTRCAT substitution model and with RAxML’s SH-like support values.

## Results

### Separate Origins of SC and Divergence From Different Subsets of *S. arcanum*

In order to quantify the extent of normal seed development within and between previously identified subgroups of *ARCA*, we performed controlled pollinations and visually scored F1 seed viability of fully developed seeds. In agreement with Rick’s (1986) classical results, we found very high proportions of hybrid seed failure (HSF) between accessions of *MARA* and all other accessions of *ARCA* (Figure 1C and Supplementary Figure 2). Moreover, near-complete HSF characterized crosses between accessions LA0378 and LA1984 (referred to herein as “*ARC-S*,” for *S. arcanum* “south”) and all other tested *ARCA*, with limited seed viability only observed in *ARC-S* × “humicho” crosses (Supplementary Figure 2). In contrast, crosses within any of these three *ARCA* subgroups yielded high proportions of normal seed development (Supplementary Figure 2). These crossing results suggest the presence of at least three subgroups within the nominal *S. arcanum* that are defined by high proportions of reciprocal HSF.

**FIGURE 2:**
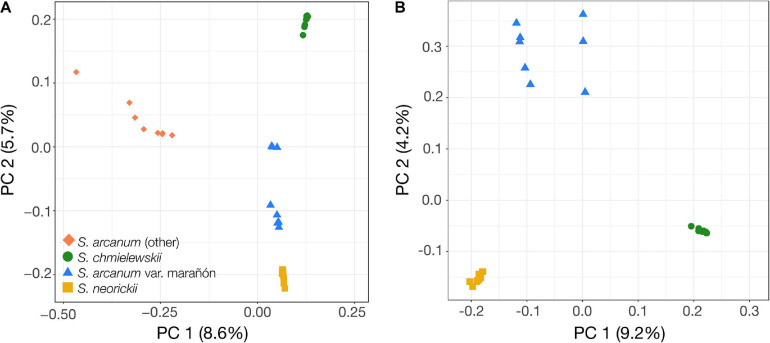
Principal Component Analyses (PCA) of genome-wide variation obtained through transcriptome sequencing. **(A)** PCA of all 39 individuals from *ARCA*, *CHMI*, and *NEOR*. PC 1 separates all non-*MARA S. arcanum* (red diamonds) from the rest, and PC 2 clearly separates *CHMI* (green dots, top), *MARA-S* and *MARA-N* subgroups of *MARA* (blue triangles), and *NEOR* (yellow squares, bottom). **(B)** PCA of 31 individuals from *MARA*, *CHMI*, and *NEOR*. Note the clear separation of “north” (*n* = 5) and “south” (*n* = 3) groups of *MARA* accessions (top left, blue triangles).

**FIGURE 3:**
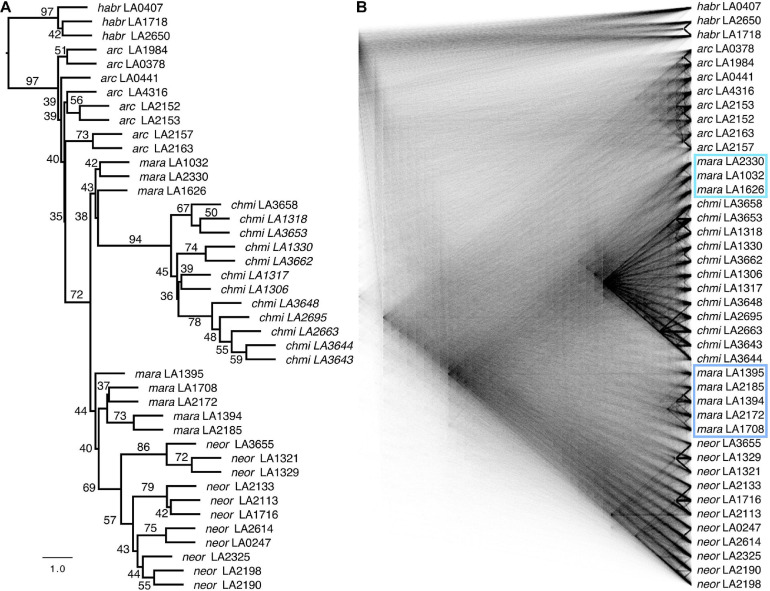
Phylogenomic reconstructions of relationships based on genome-wide SNP data. **(A)** Coalescent-based quartet-method phylogeny (ASTRAL) using 7,343 single gene trees, with quartet supports (percentage of gene trees that contain the displayed topology; see also Supplementary Figure 4). The scale bar shows branch lengths in coalescent units. **(B)** A “cloudogram” inferred from the same 7,343 single gene trees. Subgroups of *MARA* that we refer to as *MARA-S* and *MARA-N* are highlighted with a turquoise and light-blue box, respectively.

To better characterize the phylogenetic relationships within the poorly studied Arcanum species group, we used sequence data obtained from transcriptomes of individuals sampled across the geographic range of all three nominal species and performed PCA and phylogenomic analyses based on the identified variant sites (Figures 2,3 and Supplementary Figure 3). The PCA of all ingroup accessions (i.e., without *S. habrochaites*) revealed five groups, with the two *ARC-S* accessions at the extreme end of the data points for “*ARCA* (other)” along PC 1 (Figure 2A). Running PCA without the eight *ARCA* (other) confirmed the clear separation of the self-compatible species *CHMI* and *NEOR* along PC 1, but also separation of two subgroups comprising *MARA* that we refer to as *MARA-N* and *MARA-S* (for “north” and “south,” respectively; Figure 2B). Phylogenomic analyses using different methods and visualizations are fully congruent and demonstrate that *ARCA*, as currently defined, is paraphyletic (Figure 3).

As expected for rapidly diverging lineages, there is strong gene-tree discordance throughout the species tree, summarized by various low quartet-support values (i.e., close to the theoretical minimum of 1/3) and clearly visible in the gene-tree cloudogram (Figure 3B). Despite rampant gene-tree incongruence, the test of Sayyari and Mirarab (2018) revealed no evidence for any hard polytomies (Supplementary Figure 4), implying that the species tree is actually well-resolved. Both the species tree and the gene-tree cloudogram suggest a split between the ancestor of the *ARCA* (other) lineage and a clade comprising all *MARA* accessions and those of *CHMI* and *NEOR* (Figure 3 and Supplementary Figure 3). In other words, our data suggest that only *MARA*-like ancestors are strong candidates for being at the base of the extant *CHMI* and *NEOR*. Moreover, even *MARA* is inferred to be paraphyletic, with *MARA-S* being associated with the monophyletic *CHMI* and *MARA-N* being associated with the monophyletic *NEOR* (Figure 3). These patterns strongly suggest separate origins of the two self-compatible species from different ancestral populations, and thus independent paths to SC as previously inferred from their distinct defects in *S*-locus components (Markova et al., 2017).

### Modeling Speciation and Demographic Inference for Two SC Lineages

We further evaluated the ancestry and demographic history of *NEOR* and *CHMI* in the likelihood-based coalescent framework momi2 (Kamm et al., 2020). In a first step, we sought to establish the order of population splits among the two self-compatible taxa and their closest living relatives (i.e., *MARA-N* and *MARA-S*; Figure 3). Therefore, we built demographic models for all 15 possible topologies of rooted four-population trees. We note that hypotheses of simultaneous splits of more than two lineages (hard polytomies) are covered by these models, as divergence time parameters may include zero. Among the 15 alternative models, those three in which *NEOR* and *MARA-N* are sister lineages are best given our data, while the remaining 12 models are much less supported, with large increases in AIC [Table 1 (top) and Supplementary Table 4]. Parameters optimized for the best-ranking model (m6) show an early branching of *CHMI* followed by a simultaneous split of *NEOR*, *MARA-N*, and *MARA-S* (Supplementary Figure 5). Parameters optimized under the second- and third-ranking models (m2 and m15) are nearly identical, as both suggest a simultaneous split between the three populations *CHMI*, *MARA-S*, and the ancestor of *NEOR* and *MARA-N*, and similar sizes and divergence times (Supplementary Figure 5). Due to the apparent redundancy, we excluded m15 and further considered only the two top-ranking models m2 and m6.

**TABLE 1:** Comparisons of demographic models for the history of *NEOR*, *CHMI*, *MARA-N*, and *MARA-S*.

**Model**	**Tree topology**	**Bottleneck**	**Log-L**	***N* para.**	**AIC**	****Δ** -AIC**	**AIC weight**
m6	{[(maraN, neor), maraS], chmi};	None	−35688.6	10	71397.3	0	1
m2	[(neor, maraN), (chmi, maraS)];	None	−35694.4	10	71408.7	11.4	0.00332
m15	{[(maraN, neor), chmi], maraS};	None	−35743.4	10	71506.8	109.5	1.71E-24

m2.BB	[(neor, maraN), (chmi, maraS)];	neor, chmi	−35536.3	12	71096.6	0	1
m2.B2	[(neor, maraN), (chmi, maraS)];	chmi	−35543.0	11	71108.0	11.4	0.00342
m6.BB	{[(maraN, neor), maraS], chmi};	neor, chmi	−35587.9	12	71199.8	103.2	3.84E-23

*Top panel: identifying the most likely population tree topology among all 15 possible alternatives, showing the top three ranked models. Bottom panel: comparison of the two best topologies from above and six modified versions of them in which either one or both of *NEOR* and *CHMI* underwent severe bottlenecks after the split from their respective sister lineages, showing the top three ranked models. For a more comprehensive version, see Supplementary Table 4. Log-L, log-likelihood; *N* para., number of parameters; AIC, Akaike information criterion; Δ-AIC, AIC difference to best model; AIC weight, conditional probability for each model.*

In a second step, we asked how historical population size changes affect the inference of the best demographic model. In particular, we modified the two best-ranking models from the first step by introducing severe bottlenecks in either one or both of *NEOR* and *CHMI*, immediately after the split from their respective sister lineages. The size of the bottlenecks was set to *N* = 100 while their duration was a free parameter, including the possibility of zero (i.e., no bottleneck). In this comparison of eight models, the most likely one given our data yields *CHMI* and *MARA-S* as sister lineages, and bottlenecks in both *NEOR* and *CHMI* (Table 1, bottom). The introduction of simulated bottlenecks improves the model fit to the data and alters the preferred topology. Of the two simulated bottlenecks, the one at the origin of *CHMI* has a stronger effect on model fit, as all such models rank higher than models without such a bottleneck.

**FIGURE 4:**
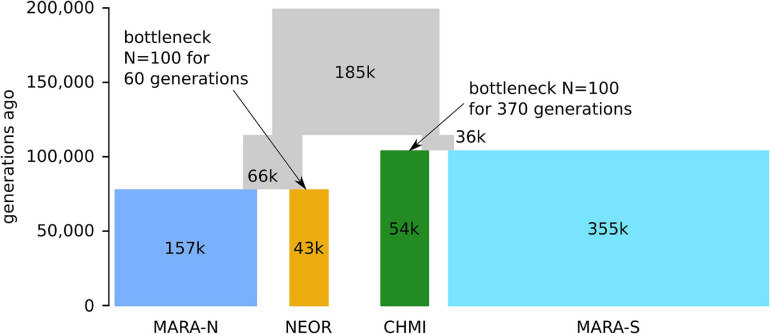
Parameter optimization under the best-fitting demographic model (m2.BB) by momi2. Time (measured in number of generations ago) and population sizes (in thousands (k) of diploid individuals) are proportional to the parameter point estimates (see Supplementary Table 5). Note that due to their short duration, the severe bottlenecks in *NEOR* and *CHMI* just after the split from their respective sister populations are not visible in this scheme.

The parameters optimized under the best model suggest that the common ancestral population of the clade comprising all studied *MARA* and the two self-compatible species diverged about 115,000 generations ago, followed shortly by another split approximately 104,000 generations ago between *MARA-S* and *CHMI* (Figure 4 and Supplementary Table 5). *CHMI* then underwent a bottleneck lasting about 370 generations, assuming a bottleneck size of *N* = 100. The second lineage descending from the common ancestral population gave rise to *NEOR* and *MARA-N* about 78,000 generations ago. Like *CHMI*, *NEOR* also passed through a bottleneck that was about sixfold milder, with approximately 60 generations at the assumed bottleneck size of *N* = 100. These modeling results clearly support the view that *NEOR* and *CHMI* are not sister lineages but rather sister to different subpopulations of *MARA* (derived from separate more recent common ancestors), and that they experienced marked reductions in effective population size (*N*_e_) after, or concomitant with, divergence.

### Patterns of Nucleotide Diversity, Absolute Divergence, and Linked Selection

**FIGURE 5:**
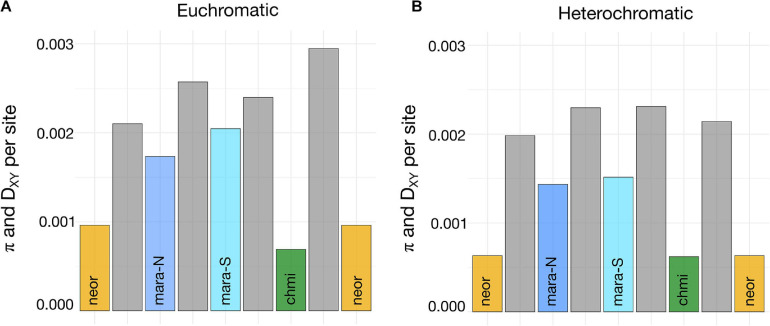
Estimates of nucleotide diversity and absolute divergence for subgroups important in the transitions from SI to SC. **(A)** Weighted estimates of π and D_XY_ across 3,103 genes representing euchromatic regions of all 12 chromosomes (high recombination; exons only); D_XY_ estimates (gray bars) are positioned between the respective π estimates (colored bars) for all comparisons. **(B)** Weighted estimates of π and D_XY_ across 619 genes from heterochromatic regions of all 12 chromosomes (low recombination; exons only). Full data are presented in Supplementary Table 6.

Levels of nucleotide diversity among species and selected subgroups were estimated from a common set of 3,722 loci that passed all our filtering steps. *CHMI* shows the lowest level of nucleotide diversity, followed by *NEOR*, *MARA-N*, and *MARA-S*; the two self-incompatible *MARA* groups harbor approximately two-to-threefold higher nucleotide diversity than the self-compatible lineages (Figure 5A). When π estimates are compared between each pair of putative closest SI relative–SC descendant, the pair *MARA-S–CHMI* exhibits a larger difference than the pair *MARA-N–NEOR* (Figure 5A), consistent with a larger drop in *N*_e_ concomitant with the origin of *CHMI* compared to *NEOR*. This reasoning assumes that levels of nucleotide diversity in the extant *MARA* groups are indicative of those at the time of divergence of the self-compatible lineages. Nucleotide diversity of the non-*MARA* group of accessions and the entire *S. arcanum* assemblage is markedly higher than for the selected lineages presented in Figure 5 (Supplementary Tables 6, 7).

Absolute sequence divergence, estimated by D_XY_, should jointly reflect time of divergence and the level of ancestral polymorphism (Cruickshank and Hahn, 2014); unlike π, it is not affected by population bottlenecks after lineage divergence. Consequently, our D_XY_ estimates for high-recombination, euchromatic regions are consistent with the most recent split being that between *MARA-N* and *NEOR*, and an earlier split between *MARA-S* and *CHMI*, while there is considerably more sequence divergence between the two self-compatible species (Figure 5A). These divergence data are congruent with the inferred topology of phylogenomic divergence, and thus independent origins of *CHMI* and *NEOR* (Figure 3).

We detected a genome-wide signal of lower levels of nucleotide diversity and divergence for loci residing within the pericentromeric, heterochromatic genome regions (Figure 5B and Supplementary Table 6). These regions comprise the majority of the tomato reference genome but are gene-poor and exhibit suppressed recombination (The Tomato Genome Consortium, 2012). Consistently lower D_XY_ values between all pairs of taxa compared to high-recombination regions imply that the fundamental differences in recombinational environment have been very stable through time, and that (most likely) selection at linked sites is responsible for reduced variation in regions of suppressed recombination (Cutter and Payseur, 2013).

Due to the reduced frequency of observed heterozygosity under partial-to-complete selfing, the effective recombination rate is reduced genome-wide, irrespective of physical recombination rate. Depending on the long-term rate of self-fertilization, selfers should thus exhibit less differences in π between eu- and hetero-chromatic regions than obligate outcrossers, as the effect of linked selection should also be apparent in regions of high recombination. While this prediction seems to be met in *CHMI* (only slightly higher π in eu- compared to hetero-chromatic regions), the differences in *NEOR* are quite substantial (compare levels of π in Figures 5A,B and Supplementary Tables 6, 7). To better compare how nucleotide diversity is distributed across the genome and how these patterns might differ in self-compatible and self-incompatible lineages, we fitted smoothed lines to the underlying diversity data for *MARA*, *NEOR*, and *CHMI*. The visualizations presented in Supplementary Figure 6 highlight the marked reduction in nucleotide diversity across pericentromeric regions in the self-incompatible *MARA*, while this trend is weaker in the self-compatible *NEOR* and almost absent in *CHMI*.

### *De novo* Assembled *SLF-23* and *S-RNase* Sequences

**FIGURE 6:**
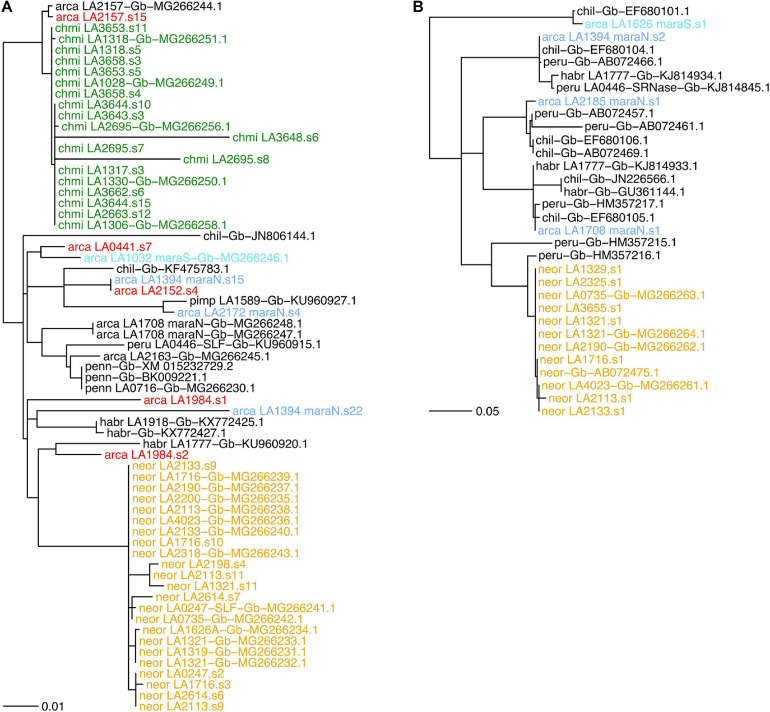
Gene trees for components of the *S*-locus in *Solanum* section *Lycopersicon*, with joint analysis of *de novo* assembled transcriptome sequences from the present study and sequences deposited in GenBank. **(A)** Gene phylogeny of the *S*-locus F-box paralog *SLF-23*. **(B)** A selected subclade for the *S*-locus *S-RNase* gene, including all *de novo* assembled sequences of *S. neorickii* with the most closely related sequences; the full *S-RNase* phylogeny is shown in Supplementary Figure 7. Gene phylogenies were estimated from coding nucleotide sequences, using midpoint-rooting. Bifurcations with less than 70% SH-like support are collapsed to polytomies. Branch lengths in number of expected substitutions per site. Group abbreviations: arca, *S. arcanum*; chil, *Solanum chilense*; chmi, *S. chmielewskii*; habr, *S. habrochaites*; neor, *S. neorickii*; penn, *Solanum pennellii*; peru, *S. peruvianum*; pimp, *Solanum pimpinellifolium*. Sequences obtained from GenBank are labeled with “Gb” and their GenBank accession number. Label colors: yellow, neor; green, chmi; light blue, *de novo* mara-N; turquoise, *de novo* mara-S; red, *de novo* “other” *S. arcanum*; black, all others.

We found between zero and two *SLF-23* sequences per sample in our transcriptomes (Supplementary Table 8). The *SLF-23* alleles closest to those from either *NEOR* and *CHMI* were found in *S. arcanum* accessions from the *ARC-S* and “chotano” groups rather than in *MARA* accessions (Figure 6A). However, *SLF-23* alleles from numerous more distantly related tomato species were placed in between those from *NEOR* and *CHMI*, emphasizing that this gene is generally in conflict with the tomato species tree, as expected for the Solanaceae *S*-locus which harbors numerous diverged haplotypes with trans-specific polymorphism (Goldberg et al., 2010; Kubo et al., 2015). Nevertheless, *CHMI* and *NEOR* exhibit reciprocal monophyly of their *SLF-23* sequences (Figure 6A), clearly supporting the notion of independent origins. We also note that *NEOR* alleles show a rather large number of substitutions compared to all other *SLF-23* sequences, raising the possibility that the ancestral haplotype (presumably derived from ancestral *MARA-N*) has not been sampled. Alternatively, this may reflect the acquisition of a functionally novel allele by gene conversion and/or duplication in *NEOR*, potentially conferring SC (Kubo et al., 2015; Markova et al., 2017).

The stylar component of the Solanaceae SI system is the hypervariable, multiallelic S-RNase, which inhibits the growth of incompatible pollen tubes. We recovered between zero and two *S-RNase* sequences from accessions of *ARCA* and *NEOR* but none from *CHMI* (Figure 6B and Supplementary Table 8). *S. neorickii*’s *de novo* assembled *S-RNase*s are nearly identical to previously reported ones and extremely homogeneous compared to alleles recovered from *ARCA* accessions. *NEOR* sequences form a monophyletic clade with respect to *ARCA*, and the most similar *de novo* sequences are all from accessions of *MARA-N* (Figure 6B and Supplementary Figure 7), consistent with the species tree.

## Discussion

Our combined crossing and molecular data help to clarify several features of the poorly understood Arcanum species complex. Previous genome-wide molecular analyses of the tomato clade did not allow definitive conclusions about the status of *S. arcanum* because only one (Pease et al., 2016) or two (Aflitos et al., 2014) plants were sequenced. In the latter case, inclusion of accessions LA2172 (*MARA*) and LA2157 (var. “chotano”) hinted at a heterogeneous assemblage and the paraphyletic nature of *S. arcanum*, but only the inclusion of more accessions across the entire group allowed us to make more robust inferences. Although the previous crossing data of Rick (1963, 1986) showing near-complete HSF between subsets of accessions were noted, they were not deemed decisive in the formal erection of *S. arcanum* as a separate species (Peralta et al., 2005, 2008). Our crossing results confirm and extend Rick’s earlier work and are consistent with our genome-wide sequence data. While the near-complete HSF between *MARA* and all other *ARCA* accessions is accompanied by marked molecular divergence, we note that the high level of HSF between *ARC-S* and the other non-*MARA* accessions has evolved with just minimal divergence (Figure 2A). This emphasizes the rapidity with which this type of postzygotic barrier can accrue (Städler et al., 2021). Below, we discuss several aspects that help to understand the origin and divergence of the two self-compatible species *CHMI* and *NEOR*, as well as the genomic consequences of these recent transitions to SC.

### Separate Origins of the Two Self-Compatible Taxa

When Rick et al. (1976) first formally described the two species *CHMI* and *NEOR*, they argued for the latter being derived from the former, mainly based on the much more conspicuous morphological selfing syndrome in *NEOR* and allozyme data showing less variation within and among populations of *NEOR*. At that time, collections of *MARA* were not yet available or had not been assessed in any depth, and thus there was no speculation on the possible origin of the two self-compatible species from outcrossing ancestors (Rick et al., 1976). With the exception of a few accessions of *CHMI*, *NEOR*, and *ARCA* as part of recent genome-wide molecular studies of species relationships (Aflitos et al., 2014; Pease et al., 2016), this group appears to not have been studied at the genome-wide level. Using equal numbers of plants from the *MARA* and non-*MARA* subgroups of *ARCA* and geographically scattered sampling, our data demonstrate that only *MARA*-like ancestors are plausible for the origin of both *CHMI* and *NEOR*, and thus the transitions from SI to SC (Figures 2, 3).

Our geographic sampling revealed the close affinity of different subgroups of *MARA* to *CHMI* and *NEOR*, with a northern group (*MARA-N*) being close to *NEOR* and a southern group (*MARA-S*) being close to *CHMI*. In other words, the current subgroup *MARA* itself appears to be paraphyletic. However, we are limited by the low number of available TGRC accessions for *MARA*, and what may appear like discontinuous groups might reflect a north–south gradient of differentiation along their natural range. We note that there is a very close correspondence of the geographic origin of accessions and their placement in our phylogenomic reconstructions (Figure 3 and Supplementary Figure 3). To confirm or elaborate on our current results would require more sampling from natural populations in Peru along the drainage of the Rio Marañón.

### Historical Inferences From Patterns of Nucleotide Diversity and Divergence

We found species-wide levels of nucleotide diversity to be lowest in *CHMI* followed by *NEOR*, and more variation in *MARA-S* than in *MARA-N*. The lower genome-wide diversity in *CHMI* compared to *NEOR* contrasts with the presumed higher outcrossing rate of *CHMI*, larger flowers with exserted stigmas (Figure 1B and Supplementary Figure 1), more allozyme diversity (Rick et al., 1976) and more sequence variation at two nuclear loci (Zuriaga et al., 2009). However, we are not aware of previous comparative studies using genome-wide markers while covering the entire species’ range. The geographic range of *CHMI* is much more restricted than that of *NEOR*, broadly overlapping with the latter’s southern range part (Figure 1A). These species are ecologically diverged to some extent (Nakazato et al., 2010) and not known to hybridize in nature, while also being reproductively isolated from *MARA* (Rick et al., 1976; Rick, 1979, 1986; Baek et al., 2016).

Levels of nucleotide diversity in high-recombination genomic regions show a roughly threefold difference in the comparison between *MARA-S* and *CHMI* and a roughly twofold difference between *MARA-N* and *NEOR*. Our raw divergence estimates (D_XY_) suggest a more recent divergence of the latter pair, at least based on high-recombination genomic regions (Figure 5). In terms of formal demographic modeling, we find strong agreement between the observed patterns of π and D_XY_ among these four groups and the inferred point estimates of the best demographic model (Figure 4). Although the current *N*_e_ of both *CHMI* and *NEOR* is estimated to be similar, the inferred ancestral bottleneck at the origin of *CHMI* was six times more severe but occurred earlier, consistent with the slightly higher D_XY_ between *CHMI* and *MARA-S* (Figure 5A).

Overall, both the observed patterns of diversity and raw divergence, as well as our demographic modeling that used other aspects of the genome-wide data, agree with a larger drop in *N*_e_ concomitant with divergence of *CHMI* compared to divergence of *NEOR*. If these inferences prove correct, the morphological changes characterizing *NEOR* (i.e., tiny flowers, no stigma exsertion; Figure 1B and Supplementary Figure 1) have been quite rapid, here inferred to be within 78,000 generations (Figure 4), which would be similar to or even faster than the rapid evolution of a morphological selfing syndrome in *Capsella*, dated at between 70,000 and 2.6 million generations (or years) ago using a mutation rate similar to our study (Sicard and Lenhard, 2011; Bachmann et al., 2019).

Across all lineages, we observed lower levels of nucleotide diversity and inter-group divergence in genomic regions of suppressed recombination (Figure 5B). This is a very common observation in population genomic studies and can most parsimoniously be attributed to the action of linked selection, where high linkage disequilibrium (LD) in regions of low recombination leads to the removal of linked, neutral variation due to both positive and negative selection on non-neutral polymorphisms (Cutter and Payseur, 2013; Cruickshank and Hahn, 2014; Slotte, 2014; Wang et al., 2016). All our D_XY_ estimates are lower in (assumed) regions of suppressed recombination, consistent with the high stability of such regions across species.

However, whether regions of low recombination will generally exhibit lower variation should also depend on gene density (a proxy for the density of targets of selection), as elegantly shown in a study on wild and domesticated rice (Flowers et al., 2012). In the tomato clade, regions of suppressed recombination are also gene-poor (The Tomato Genome Consortium, 2012), and the higher levels of nucleotide diversity for gene-dense euchromatic regions implies that despite high density of targets for selection, LD decay is rapid enough to limit the impact of linked selection, compared to regions of suppressed recombination; this is consistent with earlier data on the rapid decay of LD with physical distance in populations of self-incompatible wild tomatoes (Arunyawat et al., 2007).

### Ecological and Genomic Processes Concomitant With the SI-to-SC Transitions

Our novel demographic inferences have implications for understanding the current geographic distribution of the two self-compatible species. At face value, it seems remarkable that *CHMI*’s fairly narrow distribution in south-central Peru is far away from the likely region of origin in northern Peru (Figure 1A). We know nothing about a possibly larger geographic range of the *MARA* lineage in the past, but their current distributions are widely apart. The longer inferred time since divergence helps to make a range-shift scenario more plausible, where *CHMI* dispersed south/southeast after its divergence from the common ancestor with *MARA-S*. On the other hand, *NEOR* is partly overlapping with *MARA* in northern Peru (its presumed region of origin) but must have spread both north and south/southeast after divergence and acquisition of SC. Its current geographic distribution is fragmented and the overall range size much larger than that of its self-incompatible sister lineage *MARA-N* (Figure 1A). Range expansion is, of course, a well-known pattern after mating-system transitions from SI to SC, as exemplified by the world-wide distribution of the highly selfing *C. rubella* vis-à-vis the narrow distribution of its outcrossing congener *C. grandiflora* (Foxe et al., 2009; Guo et al., 2009).

Using geolocation data of all wild tomato species and many abiotic, mostly climatic factors, species distribution modeling suggests that *CHMI* should be able to persist in a broad zone between its current range and the presumed region of origin in northern Peru (Nakazato et al., 2010). It thus seems plausible that it has undergone a gradual range shift toward the southeast and colonized higher elevations than those typical of *MARA* populations in northern Peru. Likewise, *NEOR* now occupies an altitudinal range that is close to that of *CHMI* (Nakazato et al., 2010), while having expanded its range from the presumed region of origin in northern Peru (Figure 1A). The plausible, historical stepping-stone range shifts might have caused demographic bottlenecks and selection pressure for reproductive assurance (Busch and Delph, 2012). We suggest that the distinct patterns of genome-wide nucleotide diversity in regions of high vs. suppressed recombination can help to disentangle possible demographic and genomic contributions to the lower diversity of *CHMI* and *NEOR*.

Intriguingly, our separate analyses of π and D_XY_ across regions of normal vs. suppressed recombination revealed a pattern that is unexpected based on the inferred mating systems of *CHMI* and *NEOR*. Although (to the best of our knowledge) no formal analyses of rates of self-fertilization have ever been performed, the large, showy flowers, and exserted stigma position in *CHMI* indicate considerable potential for facultative outcrossing, while the strongly reduced size of *NEOR*’s flowers and inconspicuous inflorescences (Figure 1B and Supplementary Figure 1; Rick et al., 1976) indicate high rates of self-fertilization. Most individuals of *NEOR* set fruit autonomously in our insect-free greenhouses, while this only occurs sporadically in *CHMI* (Städler, personal observations). The inferred partial outcrossing of *CHMI* is thus at odds with the essentially flat distribution of π across the two recombination environments, contrasting with that in *NEOR* which shows markedly higher mean π in euchromatic, high-recombination regions (Supplementary Figure 6 and Supplementary Table 6), despite high inferred selfing rates.

Because the power of linked selection to remove genome-wide variation depends on the long-term rate of self-fertilization (Cutter and Payseur, 2013; Barrett et al., 2014), these genome-wide patterns can be reconciled by postulating strong demographic bottlenecks in the history of *CHMI* (removing much of the ancestral variation in euchromatic regions), whereas the more modest reductions of π in *NEOR* and its still bimodal distribution across the two recombination environments must have been shaped, at least to some extent, by linked selection. Because our formal demographic modeling assumes neutrally evolving patterns of diversity, it fails to take genomic processes such as linked selection into account. Consequently, the inferred speciation bottleneck in *NEOR* might be (at least partly) due to the action of linked selection, but the inferred much stronger bottleneck in *CHMI* seems likely to be caused mostly by demographic processes. Overall, it remains unresolved to what extent automatic selection and/or reproductive assurance (Busch et al., 2011) have contributed to these SI-to-SC transitions. Another factor that may have influenced patterns of genome-wide diversity is metapopulation dynamics, which in conjunction with a highly selfing mating system can maintain low within-population diversity but relatively high variation at the species level (Ingvarsson, 2002).

### Molecular Patterns of *S*-Locus Components Support Independent Origins of SC

Many studies with a mechanistic focus have contributed to our understanding of the S-RNase-based system of SI in the Solanaceae and its relationships to unilateral incongruity between species (Covey et al., 2010; Li and Chetelat, 2010, 2015; Baek et al., 2015; Markova et al., 2017). The pistil-expressed extracellular S-RNase recognizes “self” pollen and thus prevents self-pollination under SI; negative frequency-dependent selection maintains dozens of highly divergent alleles at this gene within populations and species. Notably, this evolutionary dynamic leads to trans-specific polymorphism, with dozens of shared *S-RNase* haplotypes across species and even genera in Solanaceae (Richman et al., 1996; Igic et al., 2006, 2007; Paape et al., 2008; Goldberg et al., 2010). One corollary of this unusual maintenance of trans-specific polymorphism is that allelic divergence does not track species divergence.

These general expectations are met in our sample of *S-RNase* sequences obtained from the self-incompatible *S. arcanum*. From some individuals, we recovered two divergent *S-RNase* sequences, while we recovered either one or no such sequences from others (Supplementary Figure 7 and Supplementary Table 8). This is expected given that we did not sequence genomic DNA but were restricted to sufficiently expressed sequences. On the other hand, it is also plausible that the absence of *S-RNase* sequences in some of our *ARCA* samples is due to highly divergent and yet unreported classes of alleles (i.e., not present among the queried diversity available in GenBank).

A very different, yet expected result is the homogeneity of *S-RNase* sequences obtained from *NEOR*, indicative of a single (once) functional *S*-haplotype that must have been derived from the ancestral population (Figure 6B). These results are fully consistent with previous work, including the finding that *NEOR* S-RNase appears to be potentially functional yet is expressed at somewhat reduced levels (Kondo et al., 2002). Likewise, our inability to recover *S-RNase* sequences from *CHMI* is consistent with previous work suggesting that several substitutions in the promoter region are likely responsible for undetectable levels of S-RNase expression (Kondo et al., 2002). The single known *S-RNase* sequence from *CHMI*, jointly with our own and other’s evidence for a single *S-RNase* allelic type in *NEOR*, adds to the common observation of much-reduced levels of allelic diversity at the *S*-locus of self-compatible species (Igic et al., 2008; Tsuchimatsu et al., 2010).

Unlike in the sporophytic SI system prevalent in Brassicaceae, in the gametophytic SI system loss-of-function mutations in the male SI/SC components lead to pollen rejection by pistil-expressed S-RNases and thus do not lead to SC (Kubo et al., 2010; Fujii et al., 2016; Markova et al., 2017). Rather, it has been proposed that SC might follow from gain-of-function mutations that allow pollen to recognize “self” S-RNase via gene duplication and/or gene conversion among *SLF* paralogs (Kubo et al., 2015), in addition to loss-of-function mutations on the pistil side. Empirical evidence in the tomato clade has documented many routes to SC that appear to be caused by loss of pistil-side SI factors, such as loss of the entire *S-RNase* gene or lack of S-RNase expression due to various sequence alterations (Kowyama et al., 1994; Royo et al., 1994b; Kondo et al., 2002; Li and Chetelat, 2015; Markova et al., 2016; Broz et al., 2017).

However, recent work in the Arcanum species group has accrued evidence for a “pistil-first” route to SC in *NEOR* (Markova et al., 2017). Specifically, these authors showed experimentally that all tested accessions of *NEOR* express both functional SLF-23 and CULLIN1 proteins in pollen, unlike their tested accessions of *CHMI*. Use of allotriploid tester lines with *S*_9_ type S-RNase (nomenclature from *Petunia*; Kubo et al., 2015) and phylogenetic analyses of published *S-RNase* sequences from *Petunia* and the tomato Arcanum group strongly suggested that the *S-RNase* allele found in *NEOR* is of *S*_9_ type (Markova et al., 2017), known to be specifically recognized by type-2 SLFs such as SLF-23 (Kubo et al., 2015; Li and Chetelat, 2015).

The collaborative “non-self” recognition model for the Solanaceae SI system posits that any functional *S*-haplotype, among its many closely linked *SLF* paralogs, cannot encode an SLF protein that recognizes the “self” S-RNase protein (Kubo et al., 2010, 2015). Any gain of an SLF protein able to recognize the “self” S-RNase, through gene duplication and/or gene conversion, is tantamount to a pollen gain-of-function mutation that would confer SC (Kubo et al., 2015). Such a switch to SC should provide selective advantages under conditions of mate limitation, such as at species’ range margins or during colonization of new habitats (Busch and Schoen, 2008; Igic et al., 2008).

While we cannot provide functional evidence regarding the investigated components of the *S*-locus, our recovered *SLF-23* sequences are consistent with the findings of Markova et al. (2017). In particular, all sequences from *NEOR* are extremely similar and markedly diverged from all *SLF-23* alleles obtained from other wild tomato species (Figure 6A), consistent with the acquisition of a functionally diverged *SLF-23* at the origin of *NEOR* and possibly its switch to SC, as advocated by Markova et al. (2017). The lack of similar sequences from our screened *MARA* accessions may also be due to low sample size and/or failed assembly. Finally, the fact that our and previously obtained *SLF-23* and *S-RNase* sequences from *CHMI* are closely related to those from the “chotano” group of *ARCA* (LA2157, LA2163; Figure 6A and Supplementary Figure 7) imply that they share a very similar *S*-haplotype, but in view of our phylogenomic evidence should not be interpreted as an ancestral–derived relationship. Rather, it is plausible that our small sample of *MARA-S* simply “missed” a corresponding *S*-haplotype.

## Data Availability Statement

Raw reads from this study are available at the SRA repository of GenBank under the BioProject accession PRJNA665625. Files associated with the demographic analyses and *S*-locus components are available at the DRYAD repository (https://doi.org/10.5061/dryad.qnk98sfg2). Other supporting data can be found in the Supplementary Tables 1–8 and Supplementary Figures 1–7.

## Author Contributions

AF-R: conceptual development, crossing study, phylogenomics, population genomics, and writing. MS: conceptual development, demographic analyses, *S*-locus analyses, and writing. MR: crossing study. TS: funding acquisition, project management, conceptual development, population genomics, and writing. All authors contributed to the article and approved the submitted version.

## Conflict of Interest

The authors declare that the research was conducted in the absence of any commercial or financial relationships that could be construed as a potential conflict of interest.

## Footnotes

1 https://tgrc.ucdavis.edu
