# Supplementary material for: Population Genomics of the “Arcanum” Species Group in Wild Tomatoes: Evidence for Separate Origins of Two Self-Compatible Lineages
Source: Front Plant Sci. 2021 Mar 19;12:624442. doi: 10.3389/fpls.2021.624442 (PMC8018279; doi:10.3389/fpls.2021.624442)
Supplement: Supplementary file 5 [file Data_Sheet_1.PDF]

Supplementary Material

1 Supplementary Data

Table S1: List of TGRC accessions with remarks (separate Excel file)

Table S2: Full seed viability data from independent crosses (separate Excel file)

Table S3: List of seed sequences for *S*-locus genes (separate Excel file)

Table S7: Per-gene estimates of π and D_{XY} for 3,722 genes (separate Excel file)

2. Supplementary Figures and Tables

Figures S1–S7

Tables S4, S5, S6, S8

2.1 Supplementary Figures

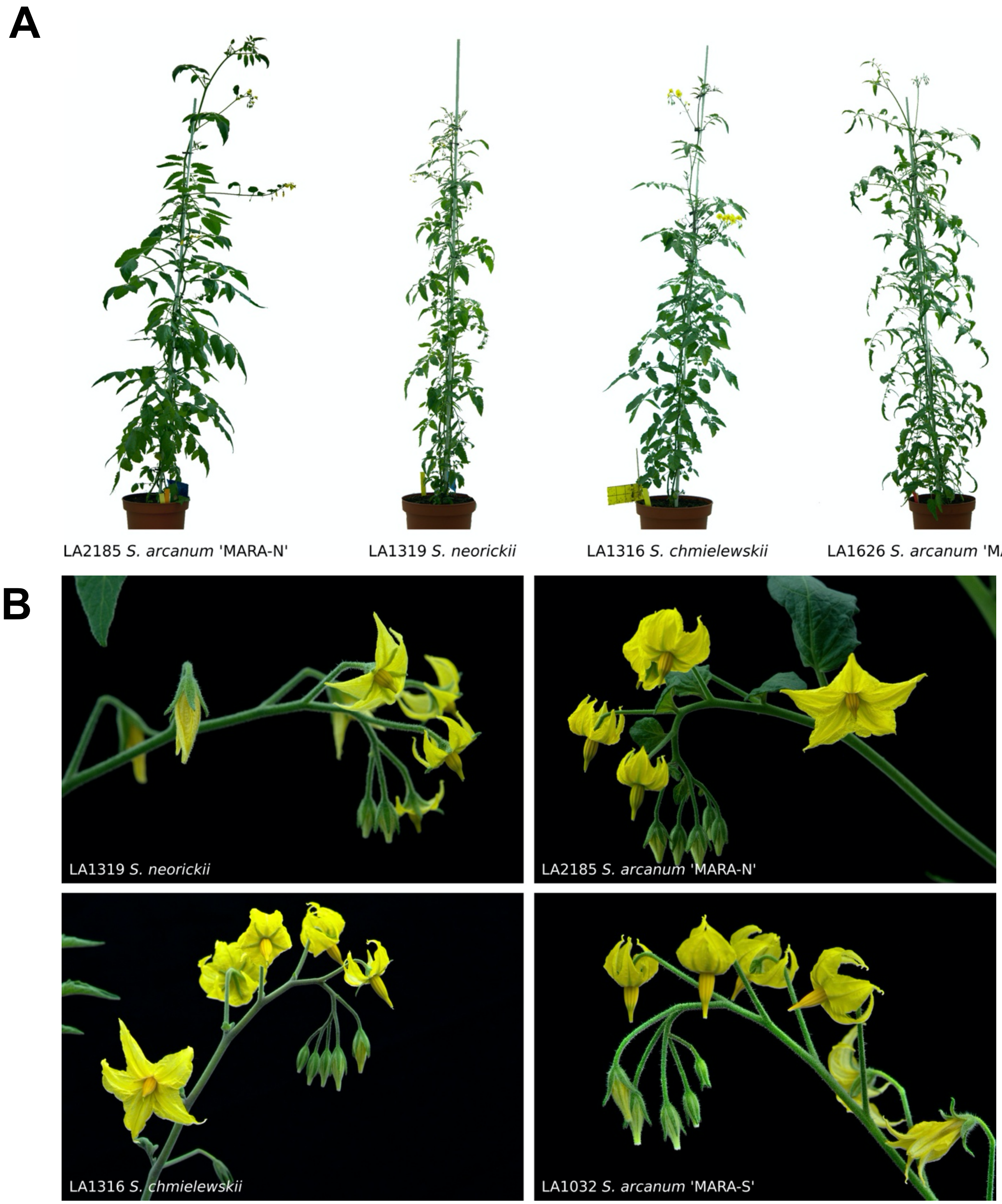

Figure S1. Whole-plant habitus (**A**) and inflorescences (**B**) of selected plants of our focal groups. Note the strong morphological selfing syndrome displayed by *S. neorickii*, e.g. much smaller flowers and a lighter tone of yellow of the corolla. *S. neorickii* plants typically show abundant fruit set due to autonomous selfing, while SI accessions need hand pollinations to produce fruit under our greenhouse conditions.

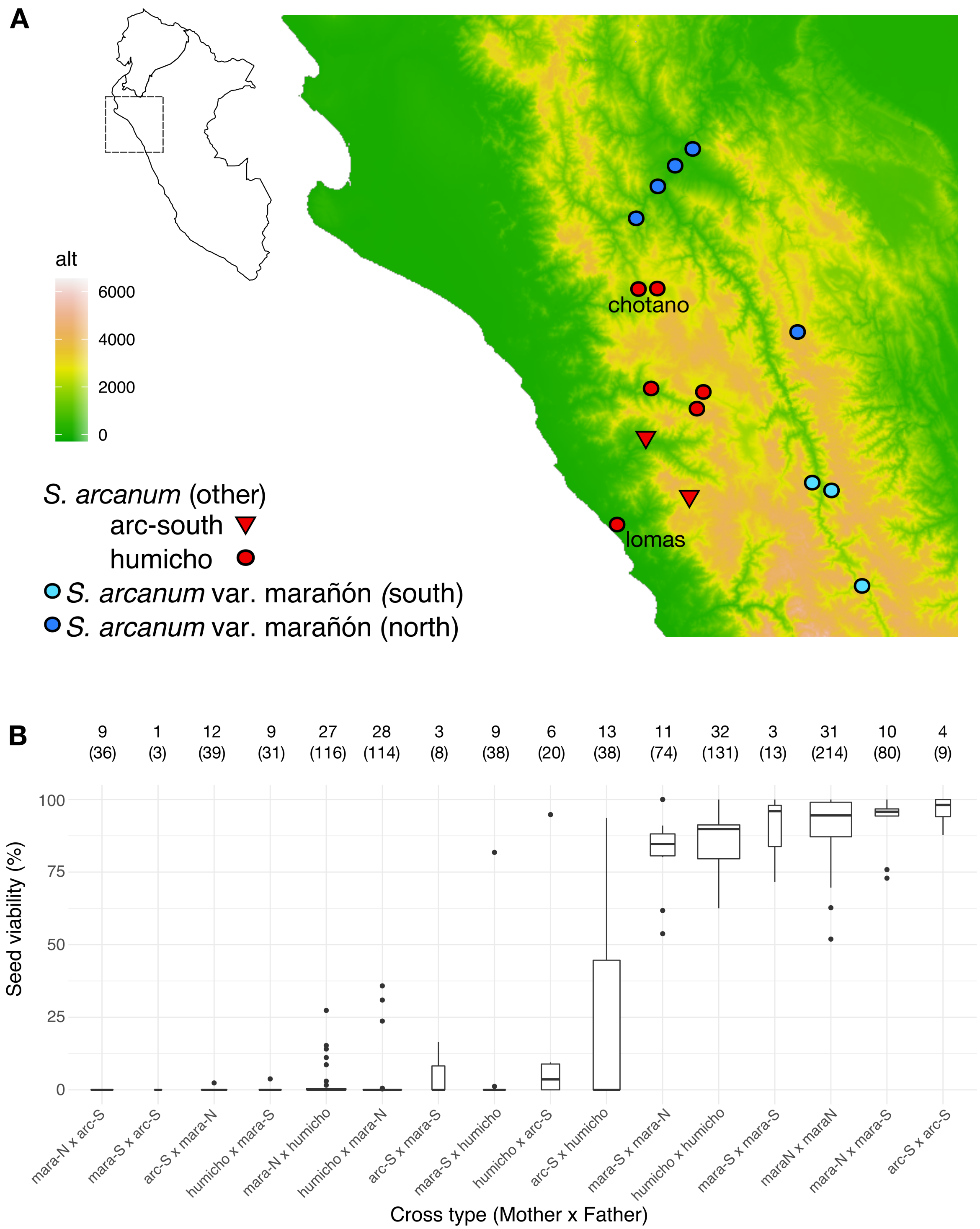

Figure S2. Map of northern Peru identifying subgroups within the nominal species *S. arcanum* (**A**), and proportion of viable seeds obtained from reciprocal crosses within and among subgroups of *S. arcanum* (*ARCA*) (**B**); 'arc-S' refers to accessions LA0378 and LA1984 from the southern range of *ARCA*; 'mara-N' and 'mara-S' refer to geographic subgroups of *S. arcanum* var. marañón (*MARA*); 'humicho' comprises the remaining *ARCA* accessions, excluding LA0441 ('lomas'). Numbers atop the graph refer to the number of independent crosses, with the number of fruits sampled in parentheses.

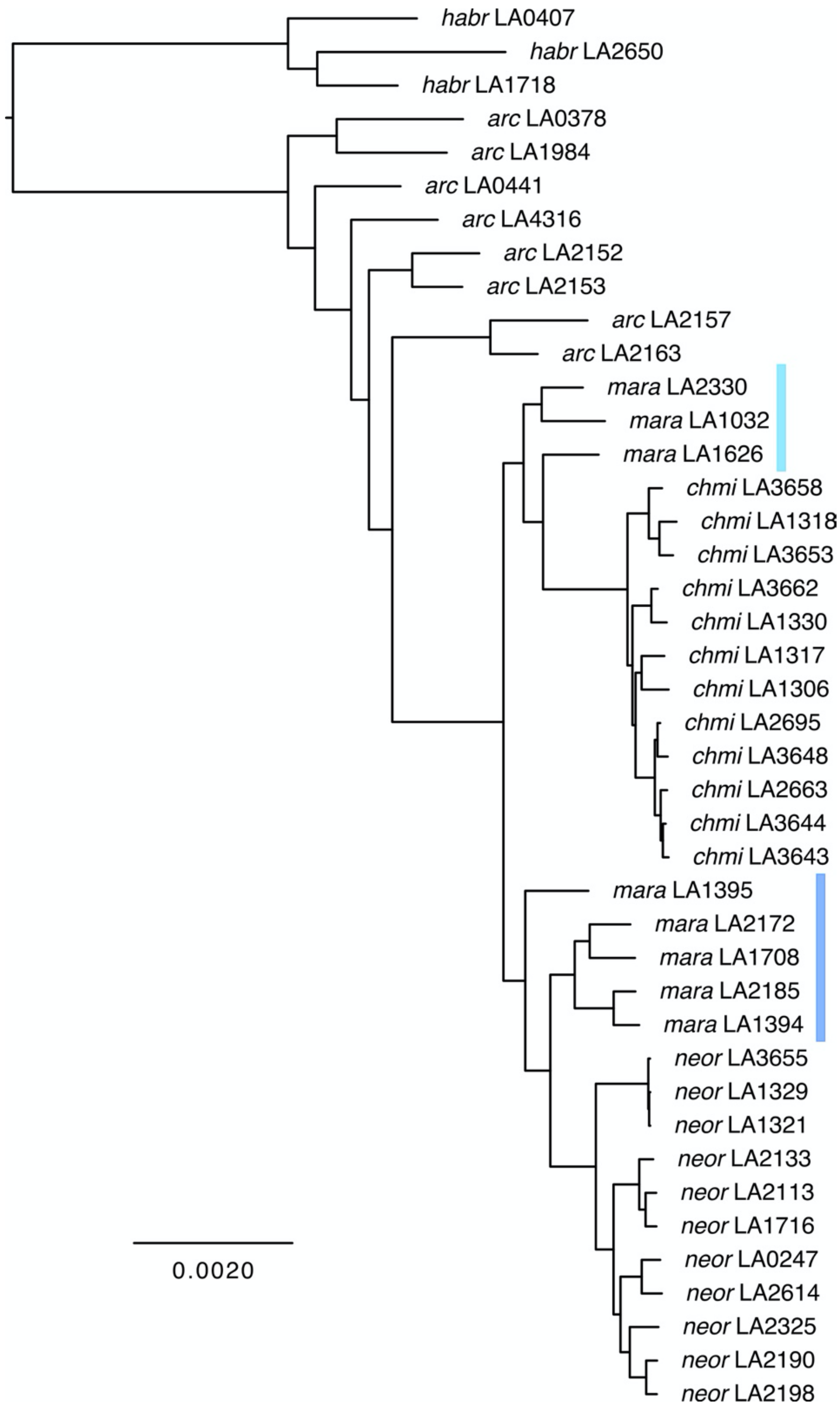

Figure S3. Maximum-Likelihood phylogenomic reconstruction of relationships based on a supermatrix of 10,829,556 aligned columns, using RAxML (Stamatakis, 2014). SH-support was 100% for all nodes. Subgroups of *MARA* that we refer to as *MARA-S* and *MARA-N* are highlighted with a turquoise and light-blue bar, respectively. Branch lengths in number of expected substitutions per site (scale bar).

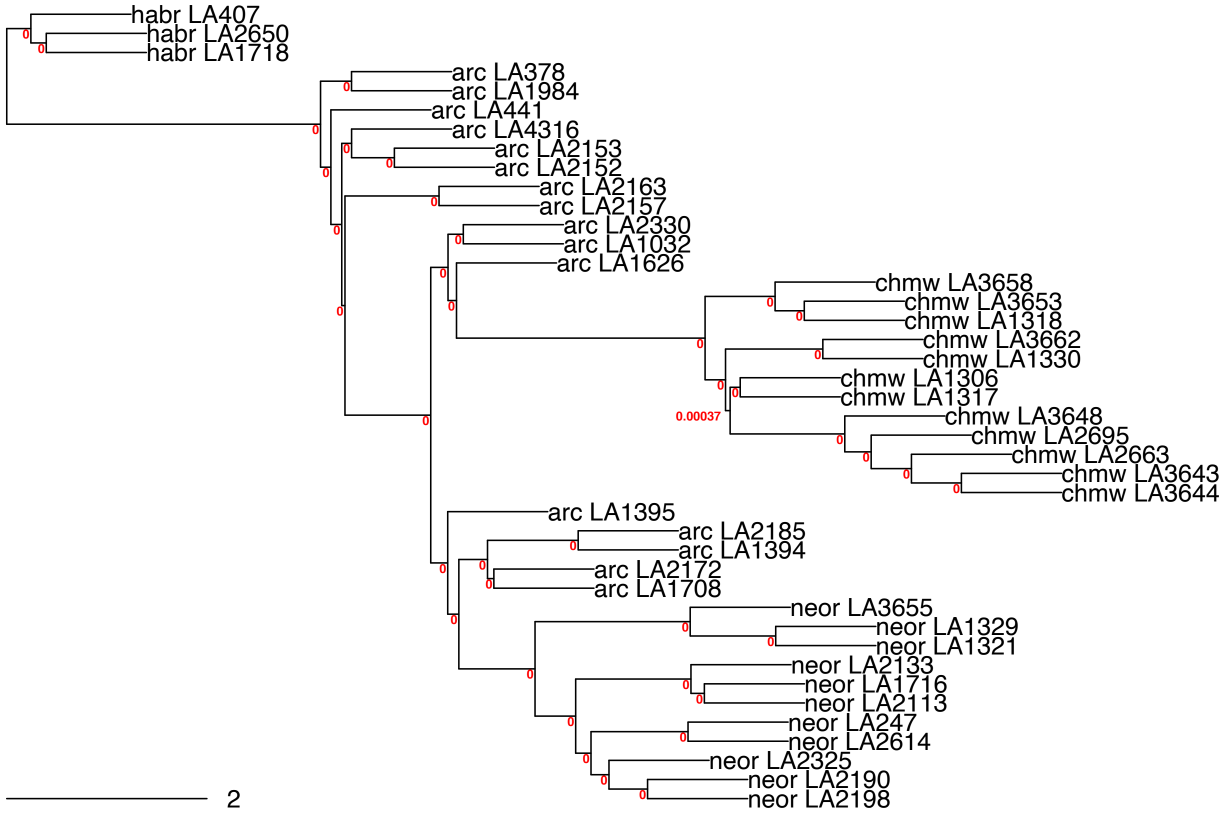

Figure S4. Coalescent-based quartet-method phylogeny (ASTRAL) using 7,343 single gene trees. *P* values resulting from applying the polytomy test (Sayyari and Mirarab, 2018) are shown along the branches (bi-partitions). Low *P* values reject the null hypothesis that a bi-partition is, in fact, a polytomy. The scale bar shows branch lengths in coalescent units.

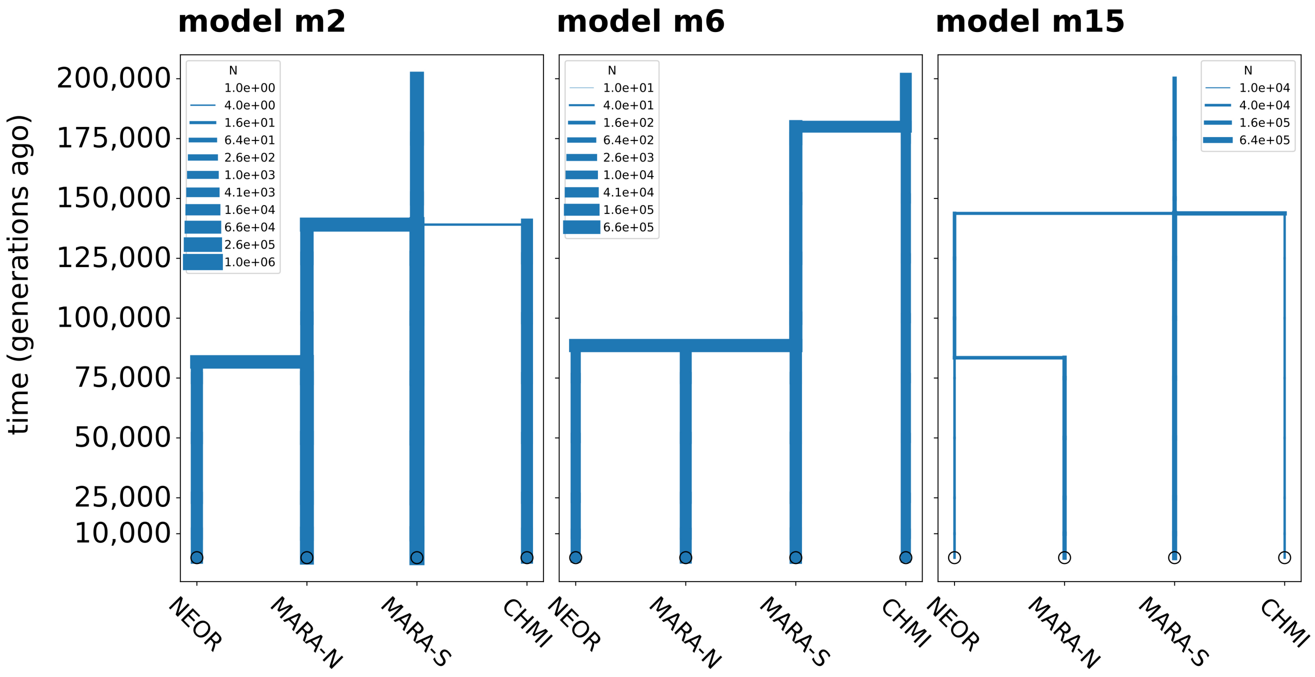

Figure S5. Overview of the three best demographic models of the Arcanum species group of wild tomatoes among 15 alternative models differing in the topology of population splits. These three models correspond to those in the upper panels of Table 1 and Table S4.

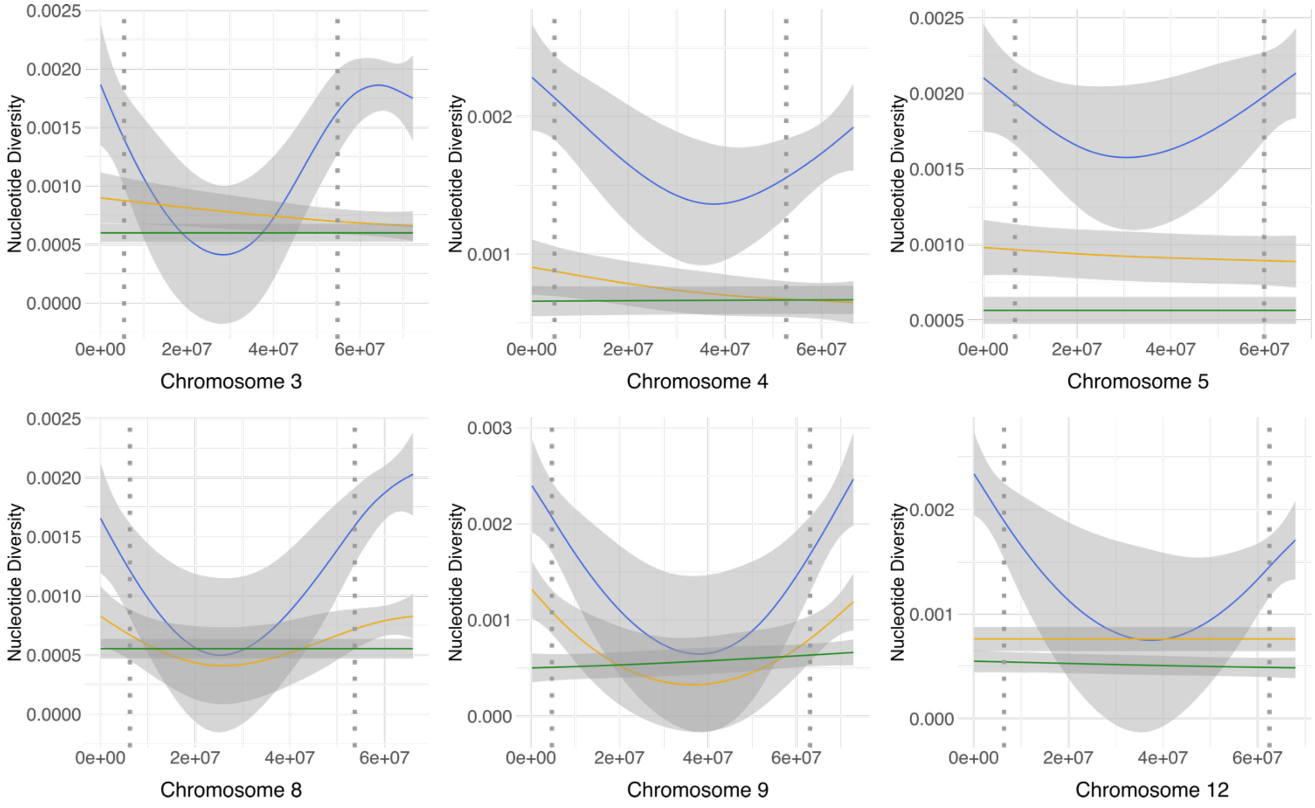

Figure S6. Illustrations of smoothed, chromosome-wide nucleotide diversity (π) for six of 12 chromosomes (3,722 genes across all chromosomes). The blue line tracks π in the self-incompatible *MARA* (8 diploid plants), the yellow line tracks π in the autogamous *NEOR* (11 diploid plants), and the green line tracks π in the self-compatible *CHMI* (12 diploid plants). Dotted grey lines roughly indicate the boundaries between euchromatic regions in the chromosome arms (high recombination rate) and the heterochromatic, pericentromeric regions (suppressed recombination) as described by Demirci et al. (2017).

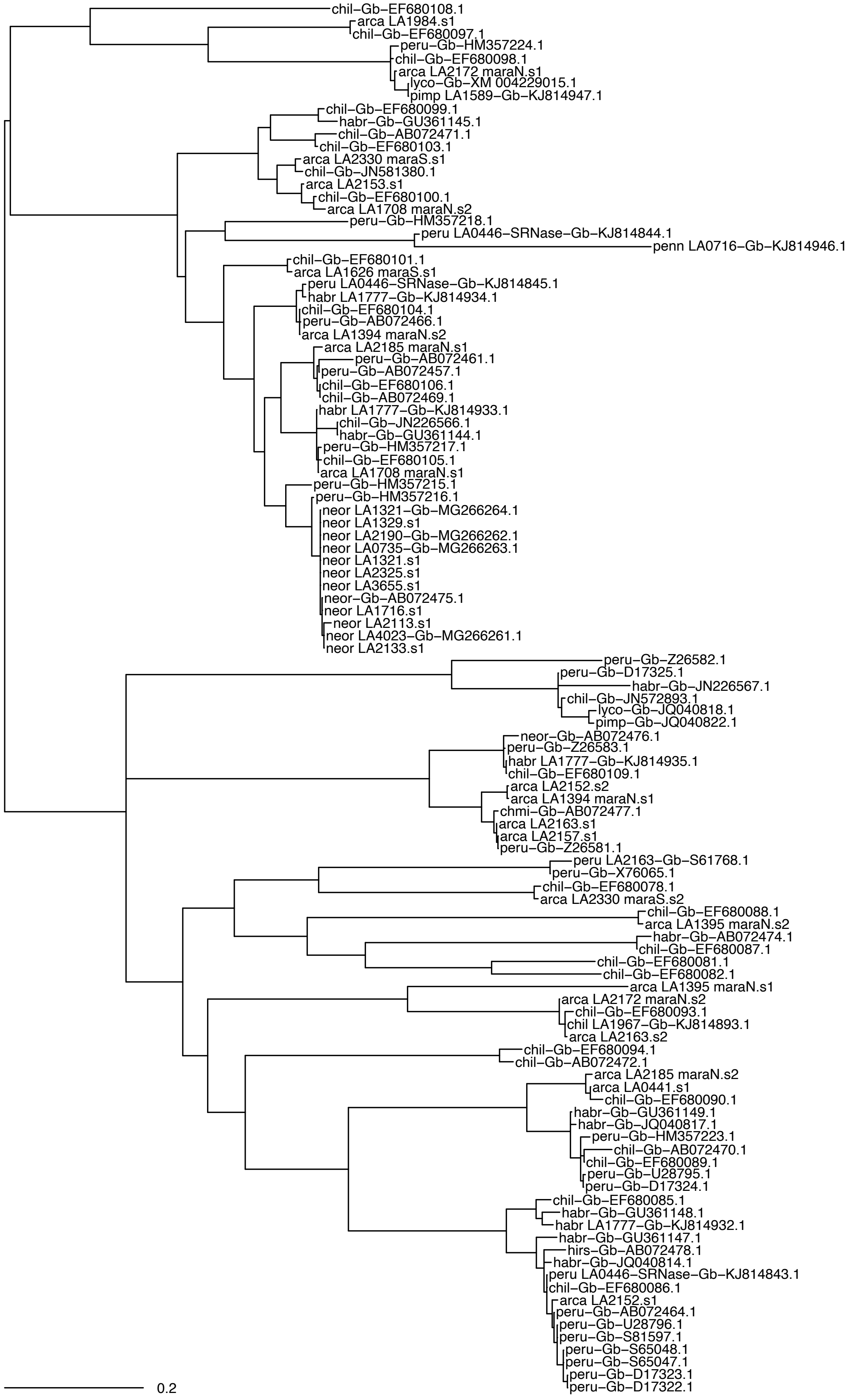
chil-Gb-EF680108.1
arca LA1984.s1
chil-Gb-EF680097.1
peru-Gb-HM357224.1
chil-Gb-EF680098.1
arca LA2172 maraN.s1
lyco-Gb-XM 004229015.1
pimp LA1589-Gb-KJ814947.1
chil-Gb-EF680099.1
habr-Gb-GU361145.1
chil-Gb-AB072471.1
chil-Gb-EF680103.1
arca LA2330 maraS.s1
chil-Gb-JN581380.1
arca LA2153.s1
chil-Gb-EF680100.1
arca LA1708 maraN.s2
peru-Gb-HM357218.1
peru LA0446-SRNase-Gb-KJ814844.1
penn LA0716-Gb-KJ814946.1
chil-Gb-EF680101.1
arca LA1626 maraS.s1
peru LA0446-SRNase-Gb-KJ814845.1
habr LA1777-Gb-KJ814934.1
chil-Gb-EF680104.1
peru-Gb-AB072466.1
arca LA1394 maraN.s2
arca LA2185 maraN.s1
peru-Gb-AB072461.1
peru-Gb-AB072457.1
chil-Gb-EF680106.1
chil-Gb-AB072469.1
habr LA1777-Gb-KJ814933.1
chil-Gb-JN226566.1
habr-Gb-GU361144.1
peru-Gb-HM357217.1
chil-Gb-EF680105.1
arca LA1708 maraN.s1
peru-Gb-HM357215.1
peru-Gb-HM357216.1
neor LA1321-Gb-MG266264.1
neor LA1329.s1
neor LA2190-Gb-MG266262.1
neor LA0735-Gb-MG266263.1
neor LA1321.s1
neor LA2325.s1
neor LA3655.s1
neor-Gb-AB072475.1
neor LA1716.s1
neor LA2113.s1
neor LA4023-Gb-MG266261.1
neor LA2133.s1
peru-Gb-Z26582.1
peru-Gb-D17325.1
habr-Gb-JN226567.1
chil-Gb-JN572893.1
lyco-Gb-JQ040818.1
pimp-Gb-JQ040822.1
neor-Gb-AB072476.1
peru-Gb-Z26583.1
habr LA1777-Gb-KJ814935.1
chil-Gb-EF680109.1
arca LA2152.s2
arca LA1394 maraN.s1
chmi-Gb-AB072477.1
arca LA2163.s1
arca LA2157.s1
peru-Gb-Z26581.1
peru LA2163-Gb-S61768.1
peru-Gb-X76065.1
chil-Gb-EF680078.1
arca LA2330 maraS.s2
chil-Gb-EF680088.1
arca LA1395 maraN.s2
habr-Gb-AB072474.1
chil-Gb-EF680087.1
chil-Gb-EF680081.1
chil-Gb-EF680082.1
arca LA1395 maraN.s1
arca LA2172 maraN.s2
chil-Gb-EF680093.1
chil LA1967-Gb-KJ814893.1
arca LA2163.s2
chil-Gb-EF680094.1
chil-Gb-AB072472.1
arca LA2185 maraN.s2
arca LA0441.s1
chil-Gb-EF680090.1
habr-Gb-GU361149.1
habr-Gb-JQ040817.1
peru-Gb-HM357223.1
chil-Gb-AB072470.1
chil-Gb-EF680089.1
peru-Gb-U28795.1
peru-Gb-D17324.1
chil-Gb-EF680085.1
habr-Gb-GU361148.1
habr LA1777-Gb-KJ814932.1
habr-Gb-GU361147.1
hirs-Gb-AB072478.1
habr-Gb-JQ040814.1
peru LA0446-SRNase-Gb-KJ814843.1
chil-Gb-EF680086.1
arca LA2152.s1
peru-Gb-AB072464.1
peru-Gb-U28796.1
peru-Gb-S81597.1
peru-Gb-S65048.1
peru-Gb-S65047.1
peru-Gb-D17323.1
peru-Gb-D17322.1
0.2

(previous page)

Figure S7. Reconstructed phylogeny for all *S*-locus *S-RNase* sequences from the tomato clade. The phylogeny was estimated from coding nucleotide sequences and midpoint-rooted. Bifurcations with less than 70% SH-like support are collapsed to polytomies. Branch lengths in number of expected substitutions per site. Species abbreviations: arca, *S. arcanum*; chil, *S. chilense*; chmi, *S. chmielewskii*; habr, *S. habrochaites*; lyco, *S. lycopersicum*; neor, *S. neorickii*; penn, *S. pennellii*; peru, *S. peruvianum*; pimp, *S. pimpinellifolium*. Sequences obtained from GenBank are labeled with 'Gb' and include their GenBank accession numbers.

2.2 Supplementary Tables

Table S4. Comparisons of demographic models for the history of *NEOR*, *CHMI*, *MARA-N* and *MARA-S*.

Model	Topology	BN?	Log-L	*N* para.	AIC	Δ-AIC	AIC weight
m6	(((maraN,neor),maraS),chmi);	none	-35688.6	10	71397.3	0	1
m2	((neor,maraN),(chmi,maraS));	none	-35694.4	10	71408.7	11.4	0.00332
m15	(((maraN,neor),chmi),maraS);	none	-35743.4	10	71506.8	109.5	1.71E-24
m12	(((maraS,chmi),neor),maraN);	none	-35924.9	10	71869.8	472.5	2.45E-103
m5	(((maraS,chmi),maraN),neor);	none	-35924.9	10	71869.8	472.5	2.45E-103
m9	(((maraS,neor),maraN),chmi);	none	-36096.9	10	72213.8	816.5	5.07E-178
m8	(((maraS,maraN),neor),chmi);	none	-36096.9	10	72213.8	816.5	5.07E-178
m10	(((chmi,neor),maraN),maraS);	none	-36163.0	10	72346.1	948.8	9.34E-207
m11	(((maraN,chmi),neor),maraS);	none	-36163.0	10	72346.1	948.8	9.34E-207
m1	((neor,chmi),(maraN,maraS));	none	-36172.4	10	72364.7	967.4	8.47E-211
m4	(((maraS,maraN),chmi),neor);	none	-36172.4	10	72364.7	967.4	8.47E-211
m3	((neor,maraS),(chmi,maraN));	none	-36175.3	10	72370.5	973.2	4.67E-212
m13	(((maraS,neor),chmi),maraN);	none	-36175.3	10	72370.5	973.2	4.67E-212
m14	(((chmi,neor),maraS),maraN);	none	-36180.7	10	72381.5	984.2	1.96E-214
m7	(((maraN,chmi),maraS),neor);	none	-36180.7	10	72381.5	984.2	1.96E-214
m2.BB	((neor,maraN),(chmi,maraS));	neor, chmi	-35536.3	12	71096.6	0	1
m2.B2	((neor,maraN),(chmi,maraS));	chmi	-35543.0	11	71108.0	11.4	0.00342
m6.BB	(((maraN,neor),maraS),chmi);	neor, chmi	-35587.9	12	71199.8	103.2	3.84E-23
m6.B2	(((maraN,neor),maraS),chmi);	chmi	-35594.2	11	71210.4	113.8	1.98E-25
m6.B1	(((maraN,neor),maraS),chmi);	neor	-35680.4	11	71382.8	286.2	7.07E-63
m2.B1	((neor,maraN),(chmi,maraS));	neor	-35686.4	11	71394.8	298.2	1.78E-65
m6	(((maraN,neor),maraS),chmi);	none	-35688.6	10	71397.3	300.7	5.03E-66
m2	((neor,maraN),(chmi,maraS));	none	-35694.4	10	71408.7	312.1	1.67E-68

Top panel: identifying the most likely population tree topology among all 15 possible alternatives. Bottom panel: Comparison of the two best topologies from above and modified versions of them in which either *CHMI*, *NEOR*, or both of them underwent severe bottlenecks after the split from their respective sister lineage. Abbreviations: BN?, bottleneck; Log-L, log-likelihood; *N* para., number of parameters; AIC, Akaike Information Criterion; Δ-AIC, AIC difference to best model; AIC weight, conditional probability for each model.

Table S5. Parameters as optimized under the best-fitting model m2.BB.

Parameter	Fitted value	2.5%	50%	97.5%
n_neor	43,191	23	42,532	48,609
n_chmi	53,715	44,637	53,551	59,001
n_maraS	355,232	266,547	351,859	426,057
n_maraN	157,413	79	157,909	180,207
n_anc1	65,756	28,544	64,574	1,907,170
n_anc2	36,095	68	41,337	5,126,777,202
n_anc3	185,139	135,033	183,342	194,575
t_merge_1	78,228	20	78,290	93,220
t_merge_2	104,318	78,078	104,208	118,142
t_merge_3	114,652	103,902	115,276	147,541
t_bottleneck_end_chmi	103,947	77,711	103,860	117,762
t_bottleneck_end_neor	78,170	3	78,224	93,220

Shown are the point estimates using the actual dataset, and the 2.5%, 50% and 97.5% quantiles of distributions obtained from parameter optimizations for 200 bootstrapped datasets. Parameter abbrevations: n_neor, population size of *NEOR*; n_chmi, population size of *CHMI*; n_maraS, population size of *MARA-S*; n_maraN, population size of *MARA-N*; n_anc1, population size of the common ancestor of *NEOR* and *MARA-N*; n_anc2, population size of the common ancestor of *CHMI* and *MARA-S*; n_anc3, population size of the common ancestor of all populations; t_merge_1, split time for *NEOR* and *MARA-N*; t_merge_2, split time for *CHMI* and *MARA-S*; t_merge_3, split time for the common ancestor of (*NEOR*, *MARA-N*) and the common ancestor of (*CHMI*, *MARA-S*); t_bottleneck_end_chmi, time of the end of the bottleneck ($N = 100$) in *CHMI* after the split from *MARA-S*; t_bottleneck_end_neor, time of the end of the bottleneck ($N = 100$) in *NEOR* after the split from *MARA-N*; all times in number of generations ago; all population sizes in number of diploids.

Table S6. Weighted means of population-genomic estimates based on 3,722 genes (weighted by the number of base pairs per gene; exonic sequences only).

**π estimates**	***ARCA***	***MARA-N***	***MARA-S***	***NEOR***	***CHMI***	***MARA***	***Humicho***
Euchromatin	0.003850	0.001734	0.002047	0.000961	0.000690	0.002194	0.003608
Heterochromatin	0.003197	0.001434	0.001515	0.000632	0.000620	0.001879	0.002544
Total	0.003752	0.001689	0.001967	0.000912	0.000679	0.002146	0.003448
**Tajima's *D***							
Euchromatin	-0.622	-0.090	-0.048	0.342	0.015	-0.362	0.066
Heterochromatin	-0.574	-0.104	0.000	0.286	0.150	-0.282	-0.118
Total	-0.615	-0.092	-0.042	0.335	0.034	-0.351	0.043
**D_{XY} (genome-wide)**							
ARCA	—	0.003160	0.003301	0.003313	0.003586	0.003214	0.003870
MARA-N		—	0.002530	0.002084	0.002734	0.001899	0.004204
MARA-S			—	0.002592	0.002384	0.002195	0.004197
NEOR				—	0.002826	0.002277	0.004136
CHMI					—	0.002604	0.004354
MARA						—	0.004203
**D_{XY} (euchromatic)**							
ARCA	—	0.003228	0.003378	0.003385	0.003664	0.003286	0.003995
MARA-N		—	0.002571	0.002102	0.002808	0.001940	0.004308
MARA-S			—	0.002646	0.002397	0.002246	0.004311
NEOR				—	0.002947	0.002309	0.004258
CHMI					—	0.002656	0.004470
MARA						—	0.004311
**D_{XY} (heterochromatic)**							
ARCA	—	0.002775	0.002864	0.002906	0.003149	0.002810	0.003163
MARA-N		—	0.002297	0.001984	0.002311	0.001668	0.003616
MARA-S			—	0.002283	0.002311	0.001910	0.003551
NEOR				—	0.002143	0.002096	0.003449
CHMI					—	0.002312	0.003701
MARA						—	0.003592

The total data set was split into euchromatic chromosome regions (3,103 genes) and heterochromatic chromosome regions (619 genes). D_{XY} and π are per-site estimates. Group names are as in the main text and have the following sample sizes (*n* diploid genomes): *ARCA* (16), *MARA-N* (5), *MARA-S* (3), *NEOR* (11), *CHMI* (12), *MARA* (8), *humicho* (6). LA0378 and LA1984 (group *ARC-S*) were excluded from the latter group (which otherwise is identical to '*ARCA* (other)' due to some divergence and partial hybrid seed failure.

Table S8. De-novo assembled *SLF-23* and *SRNase* sequences (seq.) per sample.

Sample ID	All de-novo seq.	*SLF-23* seq.	*S-RNase* seq.
arca_LA0378	0	0	0
arca_LA0441	67	1	1
arca_LA1032_mara-S	0	0	0
arca_LA1394_mara-N	35	2	2
arca_LA1395_mara-N	37	0	2
arca_LA1626_mara-S	22	0	1
arca_LA1708_mara-N	43	0	2
arca_LA1984	74	2	1
arca_LA2152	19	1	2
arca_LA2153	5	0	1
arca_LA2157	47	1	1
arca_LA2163	35	0	2
arca_LA2172_mara-N	47	1	2
arca_LA2185_mara-N	12	0	2
arca_LA2330_mara-S	23	0	2
arca_LA4316	1	0	0
chmi_LA1306	9	0	0
chmi_LA1317	20	1	0
chmi_LA1318	18	1	0
chmi_LA1330	65	0	0
chmi_LA2663	32	1	0
chmi_LA2695	14	2	0
chmi_LA3643	15	1	0
chmi_LA3644	51	2	0
chmi_LA3648	41	1	0
chmi_LA3653	46	2	0
chmi_LA3658	48	2	0
chmi_LA3662	64	1	0
neor_LA0247	24	1	0
neor_LA1321	21	1	1
neor_LA1329	19	0	1
neor_LA1716	42	2	1
neor_LA2113	44	2	1
neor_LA2133	23	1	1
neor_LA2190	15	0	0
neor_LA2198	16	1	0
neor_LA2325	23	0	1
neor_LA2614	47	2	0
neor_LA3655	107	0	1
**Sums**	**1,271**	**32**	**28**

The number of raw assembled de-novo sequences (first data column) and numbers of post-filtering retained sequences for the *SLF-23* and *SRNase* genes (second and third data columns) per sample.
